# Sex differences in pathogenesis and treatment of dyslipidemia in patients with type 2 diabetes and steatotic liver disease

**DOI:** 10.3389/fmed.2024.1458025

**Published:** 2024-09-23

**Authors:** Tatjana Ábel, Béla Benczúr, Éva Csajbókné Csobod

**Affiliations:** ^1^Department of Dietetics and Nutritional Sciences, Faculty of Health Sciences, Semmelweis University, Budapest, Hungary; ^2^János Balassa County Hospital, Ist Department of Internal medicine (Cardiology/Nephrology), Szekszárd, Hungary

**Keywords:** bempedoic acid, diabetes, ezetimibe, fibrate, MASH, MASLD, sex difference, statin

## Abstract

Previously published studies have shown that women with type 2 diabetes have a higher risk of atherosclerotic cardiovascular disease than men with type 2 diabetes. The exact reason for this is not yet known. The association between metabolic dysfunction-associated steatotic liver disease and type 2 diabetes appears to be bidirectional, meaning that the onset of one may increase the risk of the onset and progression of the other. Dyslipidemia is common in both diseases. Our aim was therefore to investigate whether there is a sex difference in the pathogenesis and management of dyslipidemia in patients with type 2 diabetes and steatotic liver disease with metabolic dysfunction. While the majority of published studies to date have found no difference between men and women in statin treatment, some studies have shown reduced effectiveness in women compared to men. Statin treatment is under-prescribed for both type 2 diabetics and patients with dysfunction-associated steatotic liver disease. No sex differences were found for ezetimibe treatment. However, to the best of our knowledge, no such study was found for fibrate treatment. Conflicting results on the efficacy of newer cholesterol-lowering PCSK9 inhibitors have been reported in women and men. Results from two real-world studies suggest that up-titration of statin dose improves the efficacy of PCSK9 inhibitors in women. Bempedoic acid treatment has been shown to be effective and safe in patients with type 2 diabetes and more effective in lipid lowering in women compared to men, based on phase 3 results published to date. Further research is needed to clarify whether the sex difference in dyslipidemia management shown in some studies plays a role in the risk of ASCVD in patients with type 2 diabetes and steatotic liver disease with metabolic dysfunction.

## 1 Introduction

Diabetes affects approximately 537 million people worldwide, which is involving one in 11 adults (1, 2). Type 2 diabetes mellitus (T2DM) is the most common form, representing 90–95% of all cases. Based on previously published studies T2DM is associated with premature atherosclerotic cardiovascular disease (ASCVD), which is the most common cause of mortality in these patients (3–5). A contributing factor is that the majority of patients with T2DM develop atherogenic dyslipidemia. Elevated triglycerides (TG) and (small dense) low-density lipoprotein cholesterol (LDL-C) levels and reduced high-density lipoprotein cholesterol (HDL-C) levels are common in patients with T2DM, even when these patients are under good glycaemic control (3–5).

Researchers in the Framingham study were the first to report in 1974 that the effect of diabetes as a cardiovascular risk factor differs between men and women (6). Men with diabetes at baseline or who were diagnosed with diabetes during the first 16 years of follow-up had a 2 times greater risk of cardiovascular death than men without diabetes, while women with diabetes had a 4.5 times greater risk than women without diabetes (6). A meta-analysis by Peters et al. showed that women with type 2 diabetes have a more than 40% greater risk of developing coronary heart disease (CHD) than men with diabetes (7). According to a meta-analysis published a few years ago, women with diabetes have a 30% higher risk of ASCVD mortality, a 58% higher risk of CHD mortality, an 8% higher risk of stroke mortality and a 13% higher risk of all-cause mortality compared to men with diabetes (8).

Steatotic liver disease (SLD) is a public health problem worldwide and increases the risk of ASCVD (9–11). Metabolic dysfunction-associated steatotic liver disease (MASLD) usually occurs in 25–45% of the general population and up to 70% of people with T2DM or ASCVD (12). MASLD and T2DM synergistically increase the risk of adverse hepatic and extrahepatic outcomes (13, 14). T2DM is an accepted risk factor for faster progression of MASLD to metabolic dysfunction-associated steatohepatitis (MASH), cirrhosis or hepatocellular carcinoma (HCC) (15). In addition, published results to date show that MASLD also increases the risk of developing and progressing to T2DM (16).

Several possible risk factors have been identified for the difference in cardiovascular event and mortality rates between women and men with T2DM, but the exact mechanism is not yet known (10, 17). Therefore, the aim of this review was to examine whether there is a sex difference in the pathogenesis and management of dyslipidemia in patients with T2DM and SLD.

## 2 Methods

The ClinicalTrials.gov, PubMed and Web of Science electronic databases were searched for full-text articles published between 1 January 2014 and 20 June 2024. The keywords used for searching were “bempedoic acid” “ezetimibe”, “fatty liver”, “fibrate”, “MASLD”, “MASH”, “NAFLD”, “NASH”, “PCSK9 inhibitors”, “Type 2 diabetes”, “sex-differences”, “steatosis”, “steatotic liver disease”, and “statin”. All results were screened for relevant articles. Authors contributed additional articles, conference abstract based on this personal knowledge.

## 3 Sex-differences in epidemiology and outcomes of MASLD/MASH

MASLD/MASH are sexually dimorphic diseases that are more common in men compared to women of reproductive age (18–20). After the menopause, the incidence of MASLD increases significantly and reaches the prevalence seen in men (21–23). In men, the prevalence of MASLD tends to increase during adulthood from young to middle age, and then decreases after the age of 50–60 years (23). In women, the prevalence of MASLD occurs about 10 years later than in men, increasing after age 50, peaking at age 60–69 and decreasing after age 70 (23). The exact aetiology of the increase in MASLD incidence in postmenopausal women is not yet clear, but age-related hormonal changes (primarily decrease in oestrogen levels), the development of other risk factors such as obesity, T2DM, hypertension, and dyslipidemia may certainly play a role (24–27).

The sex-specific occurrence of liver damage associated with the transition of MASLD to MASH and fibrosis has been less studied and showed conflicting results. Shaikh et al. investigated the risk factors for developing MASH in patients from the European cohort of the real-world GAIN study (28). Their results showed that significant covariates were age, years since diagnosis, employment status, and fibrosis stage at diagnosis, T2DM, hypertension, liver transplantation and liver biopsy at diagnosis. No significant sex-differences were found in the European cohort (28). In contrast, some studies have shown that women have a higher risk of developing MASH and fibrosis (29–31). Meanwhile, in a recent study, Tan et al. found that the age-adjusted incidence rate of MASH cirrhosis was higher in women than in men, while the age-adjusted mortality rate for women approached that of men (32).

## 4 Pathogenesis of dyslipidemia in MASLD/MASH and T2DM

The pathophysiology of MASLD is complex and includes variety of etiological factors, such as genetics, unhealthy lifestyle habits, obesity, insulin resistance, gut microbiota dysbiosis and dyslipidemia (33–39). The development of MASLD involves multiple pathogenetic molecular pathways, resulting in heterogeneity in both its pathogenesis and clinical manifestation (40–42).

About 60–70% of MASLD patients have atherogenic dyslipidaemia, characterised by high plasma TG levels, low HDL-C and elevated LDL-C (mainly small, dense LDL particles) levels (39, 43, 44). In addition, levels of TG-rich lipoproteins, such as very low-density lipoprotein (VLDL) and intermediate-density lipoprotein (IDL) are also increased.

The exact mechanism of the development and progression of MASLD in relation to impaired lipid metabolism remains unknown. The development of MASLD is due to imbalances in the transport of fatty acids (FAs) to the liver (from diet, de novo lipogenesis (DNL) and adipose tissue lipolysis), lipid synthesis and oxidation, and the hepatic export of TG as VLDL (34, 45, 46). Increased VLDL secretion and β-oxidation may compensate for increased FAs influx to the liver early in the development of MASLD (47, 48). The excessive accumulation of lipids in the liver subsequently leads to lipotoxicity, inflammation, oxidative stress and fibrosis (47, 48). The progression of MASLD to MASH causes further disruption of hepatic lipid metabolism and thus damage to liver cells.

In T2DM, the main factors underlying the development of MASLD are insulin resistance, impaired insulin secretion and abnormalities in glucose and lipid metabolism (49–51). Of these very diverse processes, differences in lipid metabolism were highlighted. Insulin resistance reduces the inhibitory effect of insulin on glucose formation and lipolysis. In adipose tissue, there is increased FAs release and de novo lipogenesis and decreased TG degradation, and in the liver decreased insulin clerarance (i.e., insulin uptake and degradation), resulting in MASLD (52, 53). Depletion of adaptive processes (e.g., activation of peroxisome proliferator-activated receptors (PPARs) further increases FAs overload, leading to a disconnection between respiration and ATP production, leading to reactive oxygen species (ROS), increased oxidative stress and ultimately the development of MASH (47).

In T2DM, persistently high blood glucose levels lead to glucotoxicity and further worsening of lipotoxicity (47, 54). These lead to increased insulin resistance, endoplasmic reticulum (ER) stress and oxidative stress, and increased inflammatory cytokine production, ultimately creating a vicious cycle (47, 54).

## 5 Sex differences in epidemiology and pathogenesis of MASLD/MASH among patients with T2DM

Obesity is often found in patients with 2TDM. Obesity increases blood levels of FFAs in both women and men. (55). However, the results so far are conflicting as to whether the two sexes are the same or whether women have higher levels of FFAs in obesity (56, 57). Although obese women have higher production of TG rich VLDL, they also have a faster rate of VLDL-TG clearance rates compared to obese men, which may contribute to the lower MASLD rate in women and lower plasma VLDL-TG levels (57, 58). Although obese women have lower TG levels compared to obese men, high TG levels in women are more closely correlated with ASCVD risk compared to men (55, 59). The exact reason for this is not known, but estrogen signaling pathways seem to play a role. (55). The hepatic estrogen signaling pathways may increase hepatic reverse cholesterol transport steps in women, thereby promoting cholesterol removal from peripherial tissues (55, 60). However, there have also been published results that found no difference between the two sexes in this regard (61).

The impact of sex-differences on the association between MASLD/MASH and T2DM incidence remains unknown. While de Ritter et al. did not show a sex-difference between the percentage of liver fat in patients with T2DM, whereas the other studies found a difference between the risk of developing MASLD/MASH in women and men with prediabetes and T2DM (25, 62–65).

Kim et al. examined the effect of sex and menopausal status on the association between MASLD and development of T2DM (62). Their results showed that the presence of MASLD, including more severe MASLD, is a stronger risk factor for T2DM in premenopausal women compared to postmenopausal women or men. It is assumed that premenopausal women with MASLD lose biological protection against risk of T2DM (62). Previous results also showed the reverse of this association, ie. younger women (< 50 years) with dysglycemia had the same risk of developing MASLD as men, compared to younger women (< 50 years) without dysglycemia, who had a lower risk (64).

In another recently published study, Succurro et al. found that women with prediabetes and T2DM have a higher OR (odds ratio) of having MASLD compared to men (25). This may explain the stronger effect of prediabetes and T2DM on MASLD in women (25). In addition, prediabetic women and women with T2DM showed significantly greater relative differences in visceral adiposity, lipid levels, homeostasis model of insulin resistance (HOMA-IR) index, and high sensitivity C-reactive protein (hsCRP) compared to prediabetic and T2DM men (25).

A recent study on the prevalence of MASH and liver fibrosis [fibrosis (F) > 2] in both men and women with T2DM has been published (65). Their results showed a significant difference between premenopausal (15.4% and 15.5%) and postmenopausal (29.5% and 30.3%) women with T2DM in terms of both MASH (*p* = 0.002) and *F* > 2 (*p* < 0.01). In contrast, no significant differences were found for MASH (*p* = 0.75) and F > 2 (*p* = 0.48) when comparing men with T2DM under 50 years (17.9% and 18.5%) and men with T2DM 50 years or older (21% and 27%).

These results support the need to detect changes in glucose homeostasis and other cardiovascular risk factors such as dyslipidemia as early as possible, especially in women.

## 6 Sex differences in the treatment of dyslipidemia in patients with T2DM and MASLD/MASH

MASLD and T2DM independently inrease the risk of ASCVD, therefore treatment of dyslipidemia in these patients is an important concern. The latest treatment guidelines for dyslipidemia recommend a more aggressive treatment for these patients to target LDL-C (66, 67).

### 6.1 Statin therapy

As reductase inhibitors of 3-hydroxy-3-methylglutaryl coenzyme A (HMG-CoA), statin therapy is a cornerstone in reducing the risk of ASCVD in people with T2DM. Statins can be given safely in individuals with MASLD without increased risk of hepatotoxicity (68, 69). This was confirmed in a recently published meta-analysis, Dai et al. found that the use of statins in the treatment of MASLD and MASH showed significant histological and biochemical improvements, particularly in hyperlipidemic patients (70). The latest clinical practice guidelines recommend statins for the treatment of dyslipidemic patients with MASLD and MASH with compensated cirrhosis (67).

According to the results of a review article and a meta-analysis including 8 randomised controlled trials have found that cholesterol-lowering statin treatment was equally effective in both sexes (71, 72). In contrast, a study by Mombelli et al. found that statin treatment improved the plasma lipid profile of dyslipidemic women with reduced efficacy compared with men (73) (Table 1). This may be partly explained by the fact that atorvastatin and simvastatin, which are predominantly metabolised by CYP3A4, that are expressed at twice the level in women than in men, leading to faster and more extensive statin metabolism and consequently lower activity compared to men (73, 74). However, the study by Mombelli et al. was retrospective and not randomized.

**TABLE 1:** Sex differences in statin therapy.

Study	Patients	Intervention	Duration	Findings
Mombelli et al. (73)	337 dyslipidemic patients (166 women; 171 men)	Different statins (atorvastatin, fluvastatin, pravastatin, rosuvastatin, simvastatin)	1 year	Statin treatment was less effective in reducing LDL-C in women (−22.7++11.8%) vs. men (−28.5+11.8%); *p* < 0.001.
Stedman et al. (77)	11.806 patients with T2DM (5184 women; 6622 men)	Different statins	10 years	Men were prescribed statins at a higher rate than women. A higher proportion of women did not reach the total cholesterol target value ( < 5 mmol/L).
Ambrož et al. (78)	10.456 patients with T2DM (47% women)	Different statins	1 year	Statin therapy was started at a lower rate among women than among men (19.7% vs. 24.7%) and prescribed less often than among men (58.7% vs. 63.9%).
Gamboa et al. (79)	4.288 patients with T2DM (55% women)	Different statins	3 years	66% of white men, 57.8% of black men, 55% of white women and 53.6% of black women treated with statin, respectively, *p* < 0.001.

For patients with MASLD, statins appear to be under-prescribed, as studies show that up to 50% of MASLD patients with an indication and 33% of MASLD patients with clinical atherosclerotic disease remain untreated (75, 76). In addition a recent study found that men with T2DM received statin therapy at a higher rate than women with T2DM, regardless of the duration of diabetes (77). Consequently, women were less likely to achieve the total cholesterol target compared to men (77). Although Stedman et al. only included patients from one region of the UK, but Salford is considered to have had a well-run diabetes service (77). Ambrož et al. investigated whether there is a sex difference in statin starting, i.e., initiation of therapy and prescribing of statins in patients with T2DM (78). Their results showed that both initiation of statin therapy [19.7% vs. 24.7%; odd ratio (OR) 0.75; 95% confidence interval (CI) 0.58–0.96] and statin prescription (58.7% vs. 63.9%; OR 0.80, 95% CI 0.73–0.89) were lower in women with T2DM compared to men. The study examined T2DM patients treated in primary care, so their results cannot be completely generalized to T2DM patients treated in other settings. Gamboa et al. investigated whether there are race or sex differences in statin use and LDL-C control in T2DM patients (79). They found that 66% of white men, 57.8% of black men, 55% of white women and 53.6% of black women used statin therapy, respectively, *p* < 0.001. These findings may also explain the sex differences in ASCVD risk in T2DM patients.

### 6.2 Ezetimibe therapy

Ezetimibe has lipid-lowering effects through its inhibition of Neimann-Pick C1-like 1, thereby reducing intestinal cholesterol absorption. However, according to the latest therapeutic guideline, the effects of ezetimibe have not yet been clearly elucidated in human large RCTs with histological endpoints for the treatment of MASLD/MASH (67).

Results from some studies have shown that women were more likely to be non-compliant with statins than men, which may have been associated with a higher rate of adverse events (80, 81). Combination therapy with statins and ezetimibe, rather than increasing the dose or intensity of statins, may be an alternative strategy for women who do not tolerate statins (82, 83). The study by Ran et al. included 93 men and 32 women with non-ST-elevation acute coronary syndrome (82). Although women and men were not tested separately in the small, short-term (3-month) study, the rosuvastatin-ezimibe combination was significantly more effective in reducing LDL-C compared with those receiving 10 mg rosuvastatin or 20 mg rosuvastatin daily. In a study by Kim et al. they observed whether there was a difference between moderate-intensity statin and ezetimibe combination or high-intensity statin monotherapy between men and women with atherosclerosis over 3 years (83) (Table 2). Their study found no sex differences in either discontinuation or dose reduction rates due to intolerance to study drugs or in the achievement of LDL-C targets in the two treatment groups.

**TABLE 2:** Sex differences in ezetimibe therapy.

Study	Patients	Intervention	Duration	Findings
Kim et al. (83)	3.780 Patients with atherosclerosis (25.2% women)	• Moderate-intensity rosuvastatin (10 mg) and ezetimibe combination or • High-intensity rosuvastatin monotherapy (20 mg)	3 years	In both sexes, significantly more patients in the combination therapy group achieved the target LDL-C level compared with the monotherapy group (*p* < 0.001). There was no difference between sexes.

### 6.3 Fibrate therapy

Fibrates are peroxisome proliferator-activated receptor alpha (PPARα) agonists, which have primarily TG-lowering and HDL-C-raising effects. In animal studies, fibrates have been effective in improving steatohepatitis and liver fibrosis, but these results have not been clearly demonstrated in human studies (84–86). The current therapeutic recommendation is that the results of studies to date are insufficient to support the use of fibrates in MASLD/MASH therapy (67). To the best of our knowledge, no study has been published that has investigated the sex-difference of fibrate treatment in patients with MASLD or MASH.

### 6.4 Proprotein convertase subtilisin/kexin type 9 (PCSK9) inhibitor therapy

Proprotein convertase subtilisin/kexin type 9 (PCSK9) inhibitors are now important lipid-lowering drugs (87–90). Alirocumab and evolocumab are fully humanised antibodies that increase the availability of LDL receptors (LDLR) on the surface of hepatocytes, leading to a reduction in LDL-C levels. Inclisiran is a small interfering ribonucleic acid (siRNA) that reduces LDL-C levels by inhibition of PCSK9 hepatic synthesis.

The results of human studies on the effect of PCSK9 inhibitors on MASLD are contradictory (91–95). Some studies have found no association, while other studies have found a beneficial effect of PCSK9 inhibitor treatment on the development and progression of MASLD (91–95).

In contrast to the results found in MASLD, a recent meta-analysis demonstrated that PCSK9 inhibitor therapy is effective and safe in patients with diabetes (96). The European Society of Cardiology in its 2023 recommendation for the treatment of dyslipidaemia in patients with diabetes recommended PCSK9 inhibitors in two groups of patients (96). Members of the first group are at very high cardiovascular (CV) risk and have persistently high LDL-C levels above target despite treatment with the maximum tolerated dose of statin in combination with ezetimibe or in patients with statin intolerance. The second group includes those who cannot tolerate statin-based treatment at any dose (even after repeated treatment) a PCSK9 inhibitor added to ezetimibe should be considered (97).

The results of sex-specific clinical trials of PCSK9 inhibitor treatment are inconclusive (98–103) (Table 3). The FOURIER (Further cardiovascular OUtcomes Research with PCSK9 Inhibition in subjects with Elevated Risk) and ODYSSEY OUTCOMES (Evaluation of Cardiovascular Outcomes After an Acute Coronary Syndrome During Treatment With Alirocumab) studies found no sex-difference in cardiovascular endpoints during treatment with the PCSK9 inhibitors (98, 99). Some results from recently published real-world studies have shown that women receiving PCSK9 inhibitor treatment had significantly lower LDL-C reduction and lower rates of LDL-C target attainment compared with men (100–103). This may be partly explained by the fact that women had higher baseline LDL-C levels than men. Paquette et al. found no difference between pre-menopausal (−58%) and post-menopausal women (−58%) in the LDL-C lowering efficacy of PCSK9 inhibitor treatment (102). It should be noted, however, that in two real-world studies, the efficacy of PCSK9-inhibitor treatment was also increased in women who received high-intensity statin combination therapy. This raises the possibility that it may be important to up titrate the dose of statins before starting PCSK9 inhibitor treatment, particularly in women (101, 102). These real-world studies, however, have various limitations, such as the Cordaro et al. study was a retrospective and observational study, the Galema-Boers et al. study was a single-centre study, and the Paquette et al. study was relatively short (100–102).

**TABLE 3:** Sex differences in PCSK9 inhibitor therapy.

Study	Patients	Intervention	Duration	Findings
Sever et al. (98) FOURIER Trial	27.564 patients with atherosclerotic cardiovascular disease	Statin and • Evolocumab or • Placebo	Median follow-up was 2.2 years	There was no difference between sexes in primary endpoints*
Bittner et al. (99) ODYSSEY Outcomes Trial	4.762 women and 14.162 men after acute coronary syndrome	Statin and • Alirocumab or • Placebo	Median follow-up was 2.8 years	Cardiovascular outcomes** were improved irrespective of sex
Cordero et al. (100)	652 patients with atherosclerotic cardiovascular disease (24.69% women)	LLT*** and • Alirocumab or • Evolocumab	2 months	The mean LDL-C reduction was 47.4% in women vs. 56.9% in men (*p* = 0.0002)
Galema-Boers et al. (101)	436 patients (209 women)	LLT *** and • Alirocumab or • Evolocumab	6 months	Relative LDL-C reduction in women compared to men was 50% vs. 61% (*p* < 0.01)
Paquette et al. (102)	259 patients (160 men, 99 women)	LLT *** and • Alirocumab or • Evolocumab	5 years	Significantly greater LDL-C response to alirocumab or evolocumab treatment in men than women (*p* < 0.0001)

*Primary endpoints were cardiovascular death, myocardial infarction, stroke, hospitalisation for unstable angina or coronary revascularisation.

**Cardiovascular outcomes were (MACE): death from coronary heart disease, nonfatal myocardial infaction, ischemic stroke or unstable angina requiring hospitalization.

***LLT, lipid-lowering therapy.

## 7 Discussion

Previous studies have shown that women with T2DM may have an excess risk of ASCVD and death compared to men with T2DM (104, 105). However, this association was confirmed not only in postmenopausal women with T2DM but also in premenopausal women (106).

The association between MASLD and T2DM is stronger than that explained by common ASCVD risk factors alone, and the relationship appears to be bidirectional (15). The association between MASLD and T2DM may also be explained by dyslipidemia (17). Most of the evidence to date suggests that postmenopausal women with T2DM have a higher risk of developing and progressing to MASLD compared to men with T2DM (65).

Therapy for MASLD is partly non-pharmacological, i.e., a lifestyle change involving the introduction of a diet and an increase in physical activity, with the main aim of weight reduction (67, 107). Therapy for MASLD may include pharmacological treatment, which also involves the management of dyslipidemia in these patients (67).

Statins are the first line treatment of dyslipidemia in T2DM and MASLD at risk of ASCVD (67, 97). A higher proportion of studies have shown that statin treatment is equally effective in both sexes, but there have also been conflicting results (71–73). It should be noted, however, that a smaller proportion of women (15–30%) participated in the larger statin trials (69). Kim et al. found no difference in the LDL-C lowering effect of ezetimibe treatment between women and men with dyslipidemia (83). With respect to fibrate therapy, to the best of our knowledge, no human studies have been found that have examined possible sex differences in dyslipidemia (83).

There are mixed results on sex-differences in recent cholesterol-lowering PCSK9 inhibitor therapy, and perhaps the dose of statin at initiation of treatment in women may be important in the effectiveness of PCSK9 treatment (98–103, 108).

It is certainly noteworthy that the first in-class inhibitor of ATP-citrate lyase, bempedoic acid therapy, has also been the subject of a phase 3 trial in T2DM patients and possible sex difference (109–111). The results showed that bempedoic acid therapy is safe and effective in patients with T2DM and greater improvements in LDL-C, and non-HDL-C and apoprotein B were found in women compared to men (110, 111). However, further studies need to evaluate the sex differences in the lipid lowering effects of bempedoic acid.

A number of questions remain unanswered, for example, is lipid-lowering therapy for women with T2DM and MASLD or MASH less effective or less aggressive than for men with T2DM and MASLD or MASH? If so, what might be the reason? Further long-term prospective studies should assess whether this difference in women with T2DM and MASLD or MASH is associated with a higher risk of ASCVD events.

This review has limitations. Patients of different ages, races, cardiovascular risk groups, with adequate or inadequate carbohydrate metabolism, and taking various blood sugar-lowering medications (SGLT2 inhibitors and GLP-1 receptor agonists reduce the risk of cardiovascular diseases) participated in the included studies. Their lifestyle (eating habits, physical activity), which is the basis of therapy for T2DM and MASLD, was often not evaluated. All of these factors made comparisons difficult and caused variability in some results.

## 8 Conclusion

Current medical recommendations to reduce the risk and mortality of ASCVD do not include sex-specific recommendations for patients with T2DM and MASLD. However, further evidence is needed which may subsequently contribute to personalized dyslipidemia therapy in these patients.

## Data Availability

The original contributions presented in the study are included in the article/supplementary material. Further inquiries can be directed can be directed to the corressponding author.
